# Population biological traits of *Periophthalmus chrysospilos* Bleeker, 1853 in the Vietnamese Mekong Delta

**DOI:** 10.7717/peerj.13289

**Published:** 2022-04-20

**Authors:** Quang Minh Dinh, Ton Huu Duc Nguyen, Tien Thi Kieu Nguyen, Tran Thi Huyen Lam, Ngon Trong Truong, Dinh Dac Tran

**Affiliations:** 1 Department of Biology, School of Education, Can Tho University, Can Tho, Viet Nam; 2 Department of Biology, An Khanh High School, Can Tho, Viet Nam; 3 Institute of High Quality Biotechnology-Food Technology, Cuu Long University, Vinh Long, Viet Nam; 4 Department of Molecular Biotechnology, Biotechnology Research and Development Institute, Can Tho University, Can Tho, Viet Nam; 5 Department Fisheries Management and Economics, College of Aquaculture and Fisheries, Can Tho University, Can Tho, Viet Nam

**Keywords:** Exploitation rate, Fishing mortality, Growth coefficient, Mudskipper, Natural mortality, Size at first capture, Total mortality

## Abstract

*Periophthalmus chrysospilos* is an amphibious fish living in mudflats from eastern India to Indonesia, including the Vietnamese Mekong Delta. Population biological traits play an important role in fishery assessment, but understanding is limited for this species. In total 1,031 specimens were caught in two regions covering four provinces, including the TVST (Duyen Hai, Tra Vinh and Tran De, Soc Trang) and BLCM (Dong Hai, Bac Lieu and Dam Doi, Ca Mau). Results found that the sex ratio was close to 1:1. The parameters of the von Bertalanffy in TVST were *L_∞_* = 12.8 cm, *K* = 0.41 yr^−1^, *t_0_* = −0.10 yr and in BLCM were 12.7 cm, 0.38 yr^−1^ and −0.08 yr, respectively. Although the growth coefficient (*Φ′*) in BLCM (1.79), was lower than that in TVST (1.83), the species shared a similar size at first capture (7.9 cm in TVST and 7.9 cm in BLCM). The species suffered from heavy pressure of fishing in TVST as fishing mortality in TVST (2.32 yr^−1^) was higher than that in BLCM (1.38 yr^−1^), leading to the higher total mortality (*Z* = 3.60 yr^−1^) in TVST compared to BLCM (Z = 2.59 yr^−1^). By contrast, the species showed similar natural mortality over both sites (1.20 yr^−1^ in TVST and 1.22 yr^−1^ in BLCM). The *Periophthalmus chrysospilos* population was reasonably exploited because *E* values (0.64 in TVST and 0.53 in BLCM) were lower than *E_10_* (0.706 in BTTV and 0.705 in STBL). Nonetheless, to avoid the consequences of overfishing, some sustainable fisheries practices should be implemented, such as protecting mangrove forests, restricting fishing during the recruitment period, using appropriate fishing tools and increasing mesh size.

## Introduction

Sustainable fishing requires a balance between the rate of exploitation and the natural regenerative capacity of stocks (Etim, King & Udo, 2002; Udoh et al., 2013). To evaluate the population stock, population biology parameters play an essential role (Al-Husaini et al., 2002). Information on both growth and mortality characteristics are also used to assess fish population biology (Amezcua, Soto-Avila & Green-Ruiz, 2006). The changes in fish growth between sex and location are regulated by growth and asymptotic length relationships (Pauly & Munro, 1984). Nevertheless, current knowledge of population dynamics is limited to *P. chrysospilos* in the Vietnamese Mekong Delta (VMD), where they are widely distributed.

Mudskippers are fish that have adapted to aerial exposure. They are active on exposed littoral areas where they actively forage (Murdy & Jaafar, 2017). *Periophthalmus chrysospilos* is widely distributed from eastern India to Indonesia (Murdy, 1989; Murdy, 2011; Murdy & Jaafar, 2017; Froese & Pauly, 2021). They can live out of water for a short period (Low, Ip & Lane, 1990; Polgar & Crosa, 2009) and are one of three species of *Periophthalmus* genus widespread in the VMD (Tran et al., 2013; Diep, Dinh & Tran, 2014; Tran et al., 2020a; Tran et al., 2020b). A potential aquarium pet, they commonly found in estuarine and coastal areas of the VMD (Tran et al., 2013; Diep, Dinh & Tran, 2014; Le et al., 2021; Tran et al., 2021). This species is a carnivore feeding mainly on shrimps *Acetes* spp. (Dinh et al., 2021b) with growth length prevailing over weight (Dinh et al., 2021c). The rainfall in the wet season is significantly higher than in the dry season (Le et al., 2006), influencing the eco-biology of *P. chrysospilos*, including its population structure. Moreover, the salinity and pH conditions in the coastal areas from TVST (Duyen Hai, Tra Vinh and Tran De, Soc Trang) to BLCM (Dong Hai, Bac Lieu and Dam Doi, Ca Mau) changed markedly. In addition, the vegetation in these two ecoregions is also different as *Sonneratia caseolaris* (L. Engl) predominates at TVST, whereas *Rhizophora apiculata* (Blume) predominates at BLCM. These factors may lead to differences in the populations biological parameters. The results of this study provide detailed information to understand the adaptability and the ecological role of *P. chrysospilos* in the VMD.

## Materials and Methods

### Study sites

**Figure 1:**
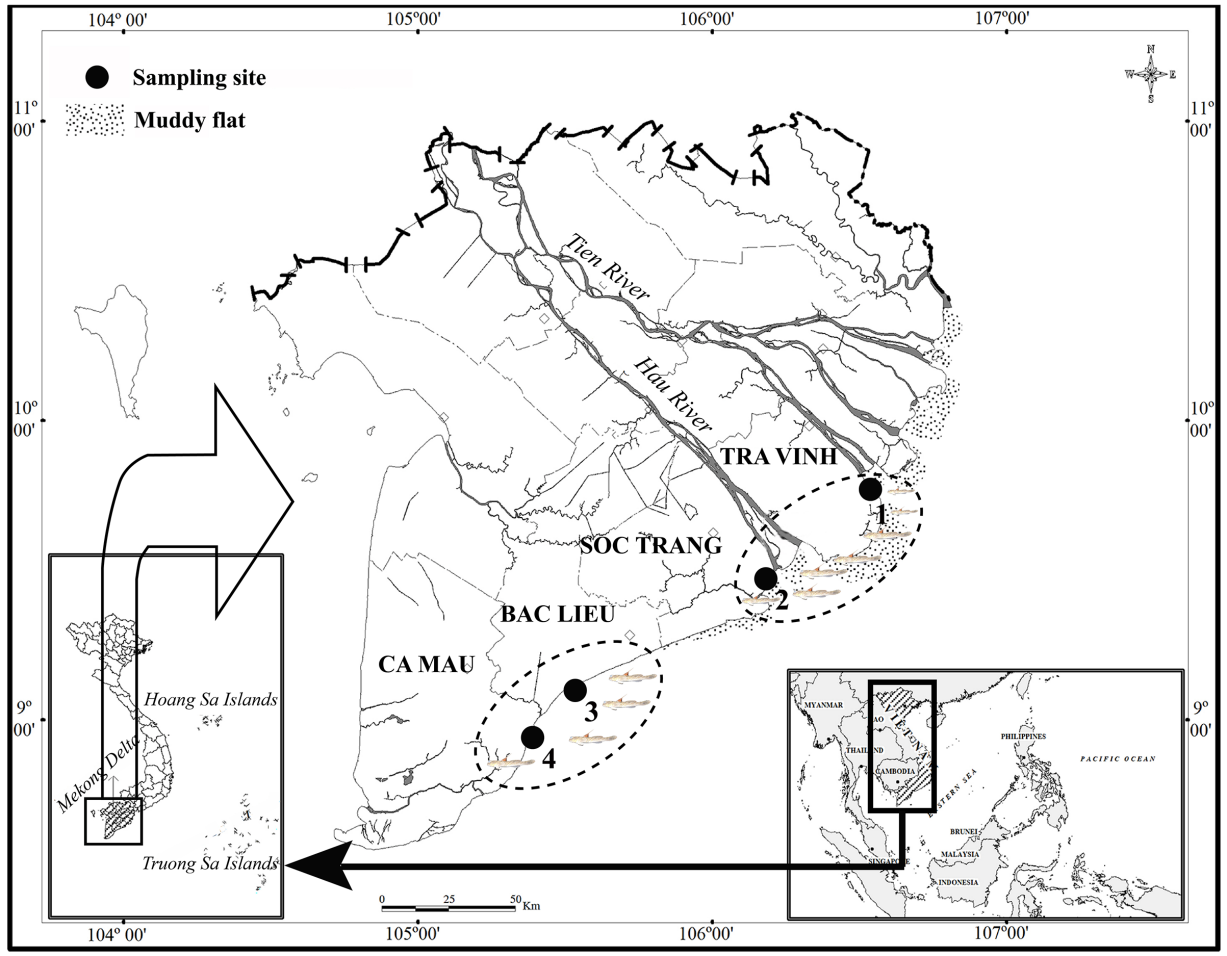
The sampling map in the Mekong Delta (modified from Dinh, 2008). (•: Sampling site; 1: Duyen Hai – Tra Vinh, 9°41′18.6″N, 106°30′35.8″E; 2: Tran De – Soc Trang, 9°29′26.8″N, 106°11′58.5″E; 3: Dong Hai – Bac Lieu, 9°06′03.2″N, 105°29′49.1″E; 4: Dam Doi – Ca Mau, 8°58′17.5″N, 105°22′51.8″E).

The study was carried out from April 2020 to March 2021 (Fig. 1). The first ecoregion consisted of two sites, Duyen Hai in Tra Vinh province and Tran De, in Soc Trang province, both of which are directly affected by the flow of the Mekong River (Co Chien estuary in Tra Vinh and Tran De estuary in Soc Trang). The second region studied included Dong Hai in Bac Lieu province and Dam Doi in Ca Mau province located in the coastal area south of the Hau River mouth. There was a slight fluctuation in temperature at each site between wet (June–December) and dry (January–May) seasons, at approximately 27 °C. Precipitation, conversely, varies significantly with ~20 mm/month in the dry season and ~400 mm/month in the wet season (Le et al., 2006). The vegetation at TVST is dominated by *Sonneratia caseolaris* and *Avicennia marina*, whereas *A. marna* and *Bruguiera gymnorrhiza* (L.) Savigny occurs predominantly at BLCM. The salinity in BLCM (23.2–23.5) was higher than that in TVST (10.4–12.3), whereas the reverse case was found for pH (pH is 7.8–7.9 in TVST and 7.6–7.7 in BLCM, respectively) (Dinh et al., 2021a).

### Fish collection and analysis

Fish samples were collected using the method of Dinh et al. (2021b). Accordingly, fish were caught every month in both eco-zones by hand-catching at dusk in mudflat areas of 120 square meters (6 m × 20 m). Fishes were identified following Murdy & Jaafar (2017) and sexed. The first dorsal fins of females were shorter, smaller, and less colourful than that of males. The genital papillae of females were bulbous and pinkish, and equally broad at the base and tip, whereas those for males was slender and whitish, broad at the base and tapered towards the tip (Dinh et al., 2020b). After catching, fishes were immediately anaesthetized in a solution of tricaine methanesulfonate (MS222) and transferred into a solution of 5% formalin (Animal Welfare Assessment No. BQ2020-03/KSP). Specimens were then transferred to the laboratory to determine the fish total length (*L*) to the nearest 0.1 cm.

### Data analysis

The *χ*^*2*^ was used to verify if the male to female ratio differed from 1:1 ratio (Zar, 1999). The population parameters were analyzed by the FiSAT II software based on length-frequency (Gayanilo, Sparre & Pauly, 2005).

First, the asymptotic length (*L*_*∞*_) and the growth parameter (*K*) values were obtained from the ELEFAN I procedure (Pauly & David, 1981; Pauly, 1982; Pauly, 1987). Whilst, the theoretical age parameter (*t*_*0*_) was inferred from the equation: Log_10_(*−t*_*0*_) = −0.3922 to 0.2752log_10_*L*_*∞*_ − 1.038log_10_*K* (Pauly, 1979).

Second, the natural mortality (*M)* was calculated by the mathematical expression (Pauly, 1980): Log*M* = −0.0066 to 0.279Log*L*_*∞*_ + 0.6543Log*K* + 0.463Log*T* where: *L*_*∞*_ and *K* from ELEFAN I, and T was the mean annual water temperature (°C). Meanwhile, the total mortality (*Z*) was estimated from length-converted capture curve data (Beverton & Holt, 1957; Ricker, 1975). The fishing mortality (*F*) was calculated by subtracting the total mortality (*Z*) from the natural mortality (*M*). The ratio between *F* and *Z* was the exploitation rate (*E*) (Ricker, 1975).

Third, the length-converted catch curve of fish was obtained by comparing the cumulative capacity graph of capture and to the mid-length of the population. For example, *L*_*c*_ was the length at which fish was ready to be caught (Pauly, 1987). Then, the length-converted catch curve calculates the catching capacity of each fish size class. The stock and yield of this fish species were estimated from the model documented by Beverton & Holt (1957).

Finally, the maximum exploitation rate (*E*_*max*_), the optimized exploitation rate (*E*_*0.1*_), and the exploitation rate of 50% stock reduction (*E*_*0.5*_) was obtained from the knife-edge selection procedure (Beverton & Holt, 1966). Fishing status was assessed based on the value of the isopleth (*L*_*c*_*/L*_*∞*_) (Pauly & Soriano, 1986). The von Bertalanffy growth performance (*Φ′ = LogK + 2LogL*_*∞*_), as demonstrated by Moreau, Bambino & Pauly (1986), was applied to obtain the growth rate according to sampling site.

## Results

A total of 1,031 individuals of *P. chrysospilos* (523 males and 508 females) were caught from the two ecoregions (Table 1). The number of males was lower in the estuarine ecological region (TVST) than that of females (240 males and 259 females). Meanwhile, more males were caught in the BLCM than females (283 males and 249 females). The sex ratios for caught fish in both ecoregions was approximately 1:1 (*χ*^*2*^= 0.22, *p* > 0.05).

**Table 1:** The number of *Periophthalmus chrysospilos* from April 2020 to March 2021.

Month	Total length (cm)								
	3–4	4–5	5–6	6–7	7–8	8–9	9–10	10–11	Total
Apr-20	1	9	21	29	22	8	1	2	93
May-20		1	39	17	14	4	3		78
Jun-20		1	7	10	51	37	9	1	116
Jul-20		2	3	4	22	64	15		110
Aug-20		2	4	18	21	26	9		80
Sep-20		3	4	11	32	41	7		98
Oct-20			3	11	10	26	14		64
Nov-20		2	10	16	18	19	7	1	73
Dec-20		2	12	18	5	31	13	1	82
Jan-21			16	9	1	23	30	2	81
Feb-21			18	14	4	11	24	3	74
Mar-21	1	8	9	13	29	21	1		82
Total	2	30	146	170	229	311	133	10	1,031

**Figure 2:**
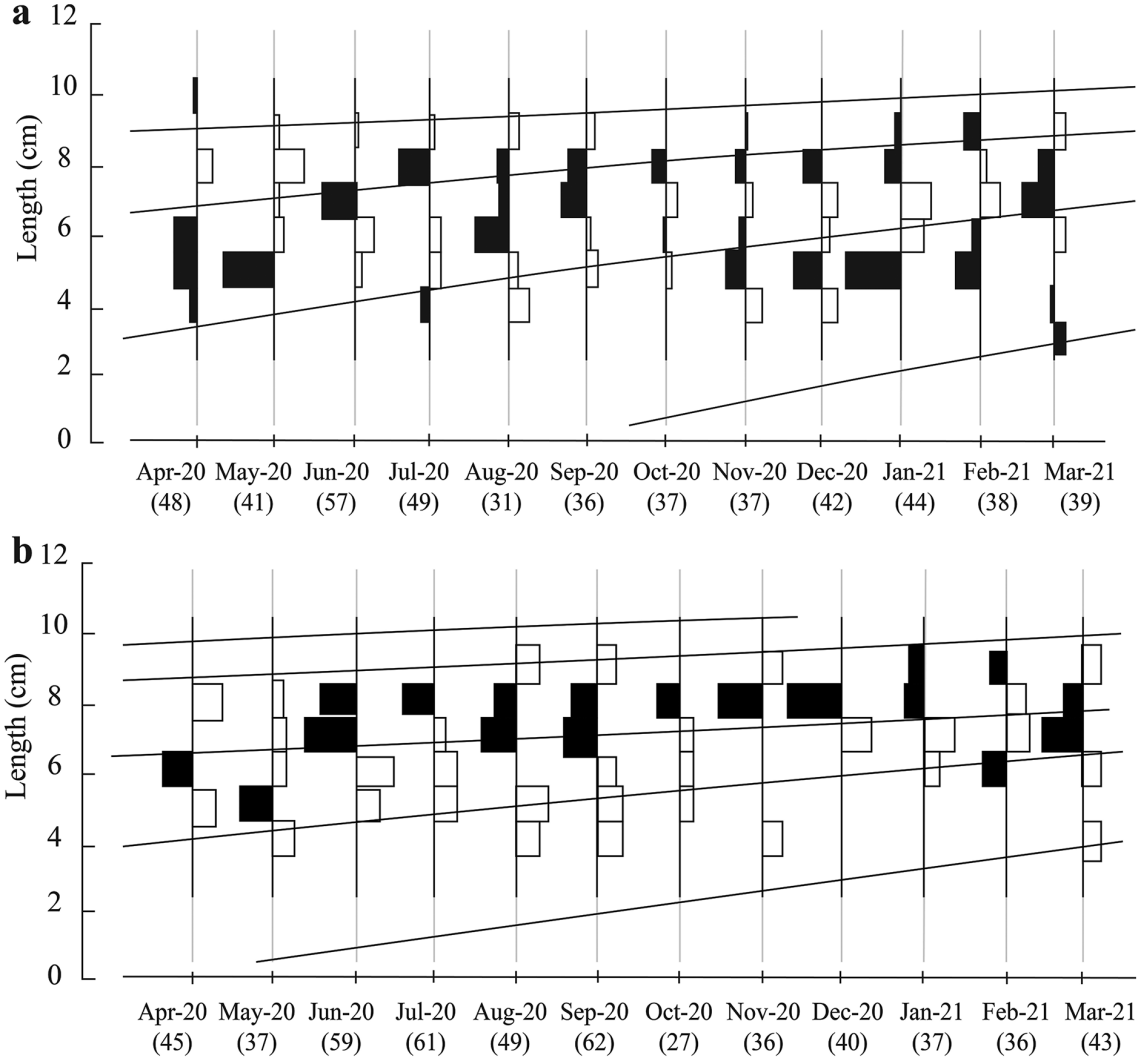
Growth curves of *Periophthalmus chrysospilos* estimated by means of ELEFAN I superimposed on restructured length-frequency data in TVST (A, *n* = 499) and BLCM (B, *n* = 532). Here length-frequency data corrected for gear selection (Pauly & Soriano, 1986) are presented. These graphs’ black and white bars represented the fluctuation of average length groups over the months. TVST: Duyen Hai, Tra Vinh and Tran De, Soc Trang; BLCM: Dong Hai, Bac Lieu and Dam Doi, Ca Mau.

The total fish length ranged in the two ecoregions from 3.0 to 10.6 cm. Here, the most popular length classes were 7.0–8.0 and 8.0–9.0 cm (Fig. 2). There were four groups of different ages in TVST (Fig. 2A). However, the length frequency values showed that at BLCM, the fish population had five different age groups (Fig. 2B).

**Figure 3:**
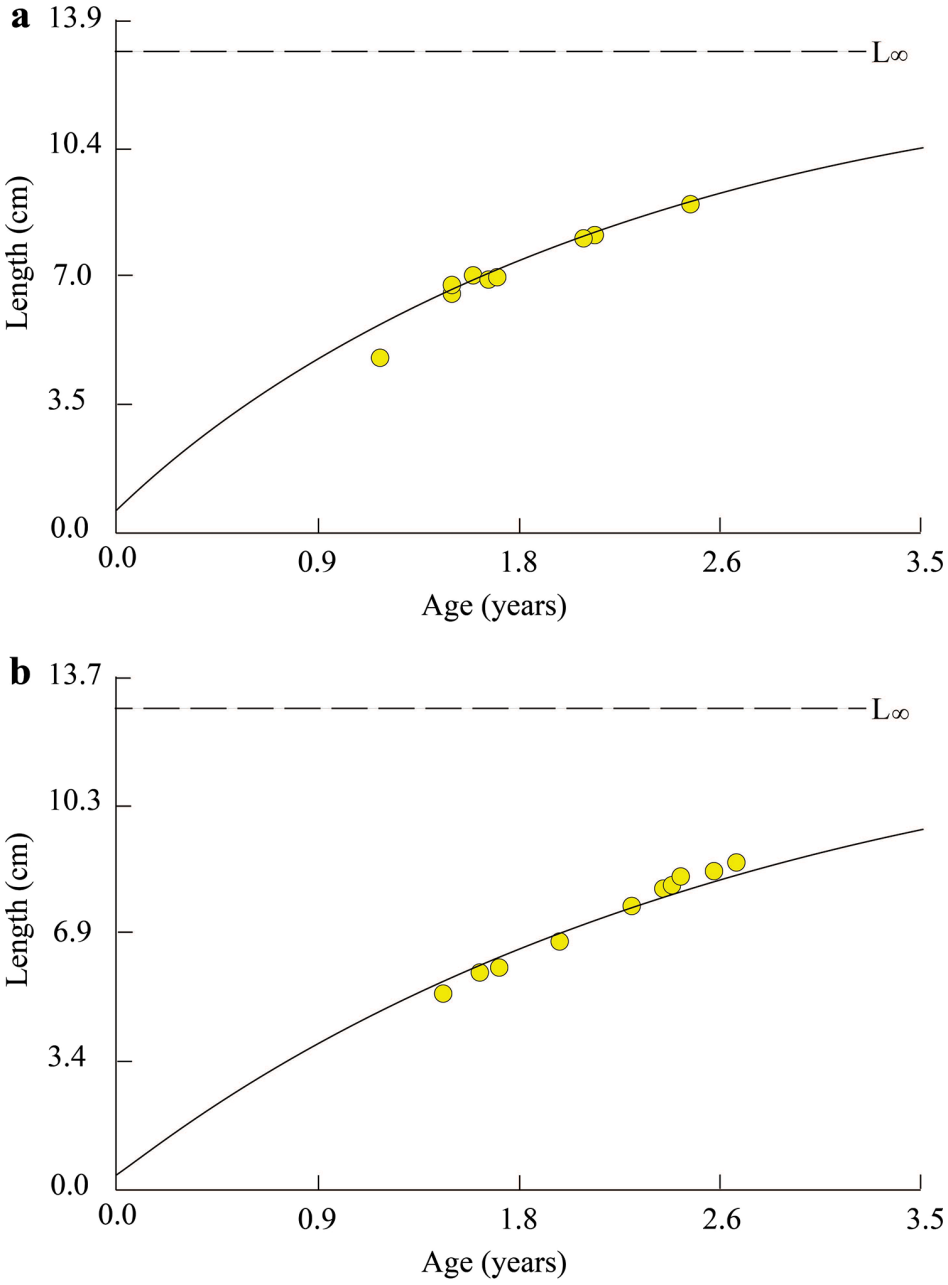
Growth curve of *Periophthalmus chrysospilos*. (A) Duyen Hai, Tra Vinh and Tran De, Soc Trang; (B) Dong Hai, Bac Lieu and Dam Doi, Ca Mau.

At TVST, the von Bertalanffy curve of *P. chrysospilos* population was *L*_*t*_ = 12.8(1 − *e*^*−*0.41 (*t* + 0.10)^) (Fig. 3A). This curve in the BLCM was *L*_*t*_ = 12.7(1 − *e*^−0.38 (*t* + 0.08)^) (Fig. 3B). These parameters in BLCM were lower than that in the TVST ecoregion. Almost all fish were between 1.0 and 2.6 years old in TVST and from 1.5 to 2.8 years of age in BLCM. The *t*_*0*_ value in the BLCM revealed the eggs of fish hatched earlier (0.8 month) than in the TVST area (1 month).

**Figure 4:**
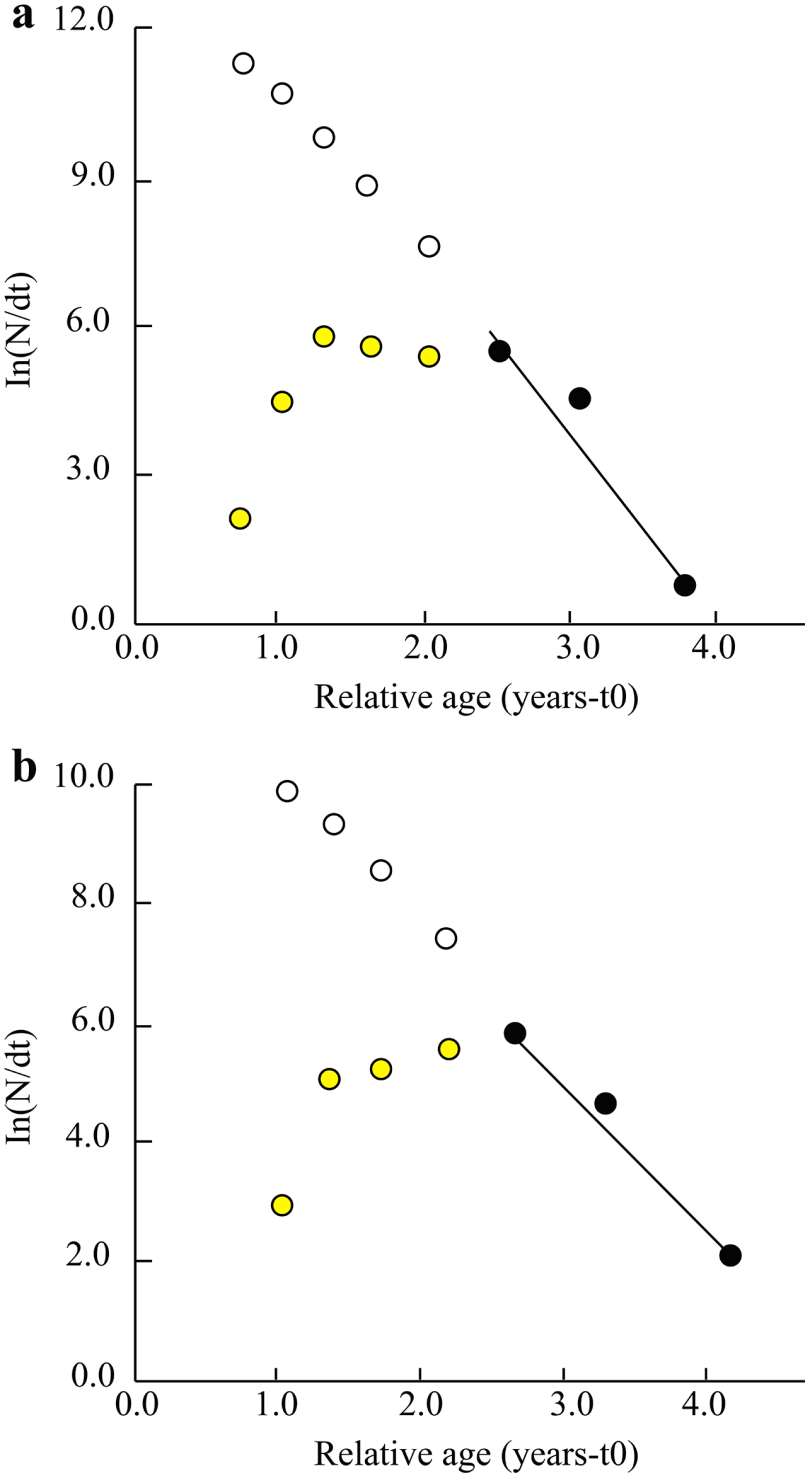
The length converted catch curve of *Periophthalmus chrysospilos*. (A) Duyen Hai, Tra Vinh and Tran De, Soc Trang, *Z* = 3.60 yr^−1^, *M* = 1.20 yr^−1^, *F* = 2.32 yr^−1^, *E* = 0.64; (B) Dong Hai, Bac Lieu and Dam Doi, Ca Mau, *Z* = 2.59 yr^−1^, *M* = 1.22 yr^−1^, *F* = 1.38 yr^−1^, *E* = 0.53; yellow data points: not used; dark data points: used; open circles indicate predicted abundance of fish in the partially selected size classes.

Other parameters such as total mortality (*Z*), natural mortality (*M*), fishing mortality (*F*) and exploitation ratio (*E*) in the TVST ecoregion were 3.60/years, 1.20/years, 2.32/years and 0.64, respectively. (Fig. 4A). Similarly, at BLCM, these parameters were *Z* = 2.59/years, *M* = 1.22/years, *F* = 1.38/years and *E* = 0.53 (Fig. 4B).

**Figure 5:**
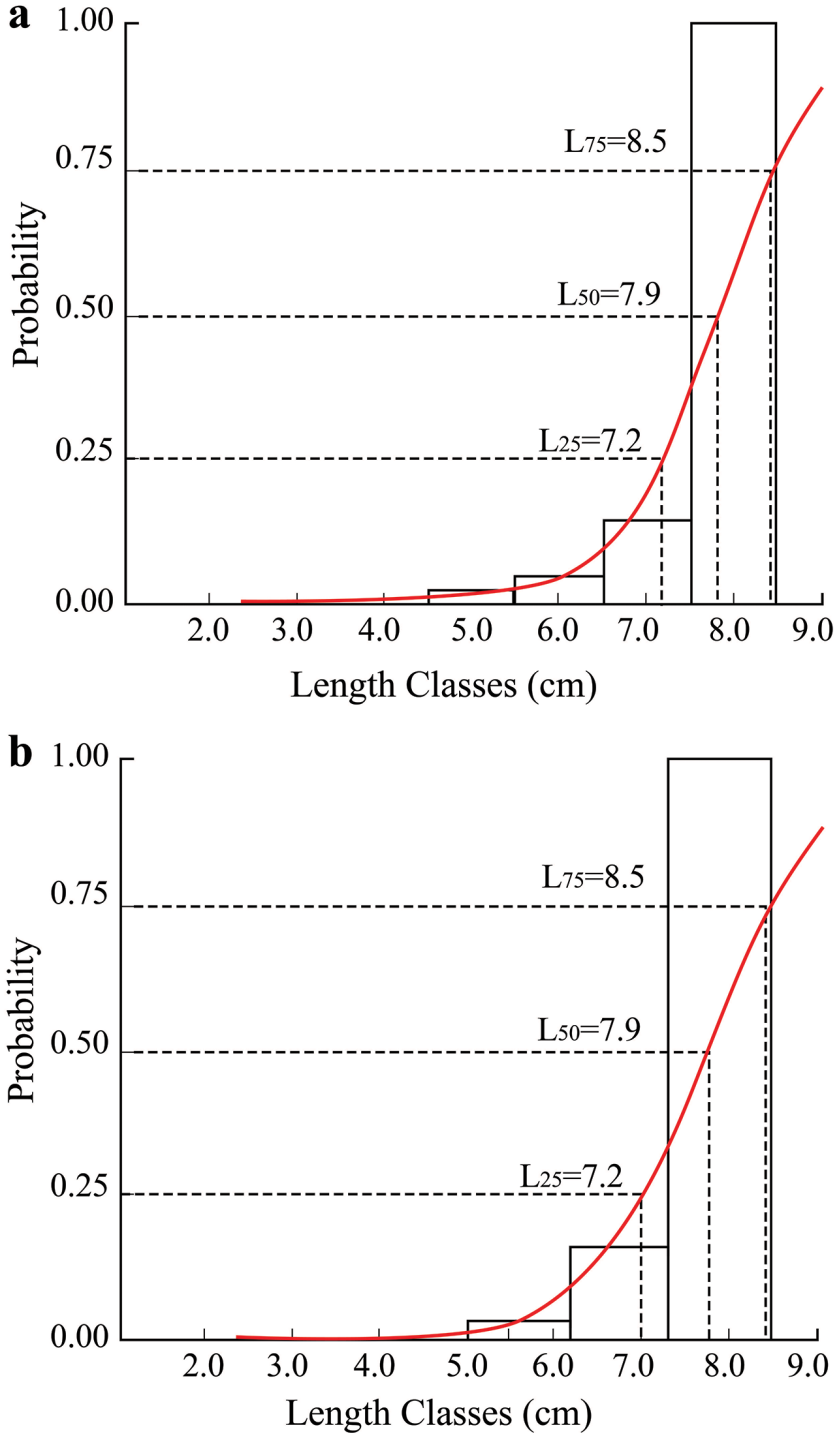
The length converted catch curve of *Periophthalmus chrysospilos*. (A) Duyen Hai, Tra Vinh and Tran De, Soc Trang; (B) Dong Hai, Bac Lieu and Dam Doi, Ca Mau.

In the TVST area, fish had a maximum length of 12.8 cm, and the first caught length (*L*_*50*_) was 7.9 cm. In the BLCM region, the first caught length was also 7.9 cm (Fig. 5).

**Figure 6:**
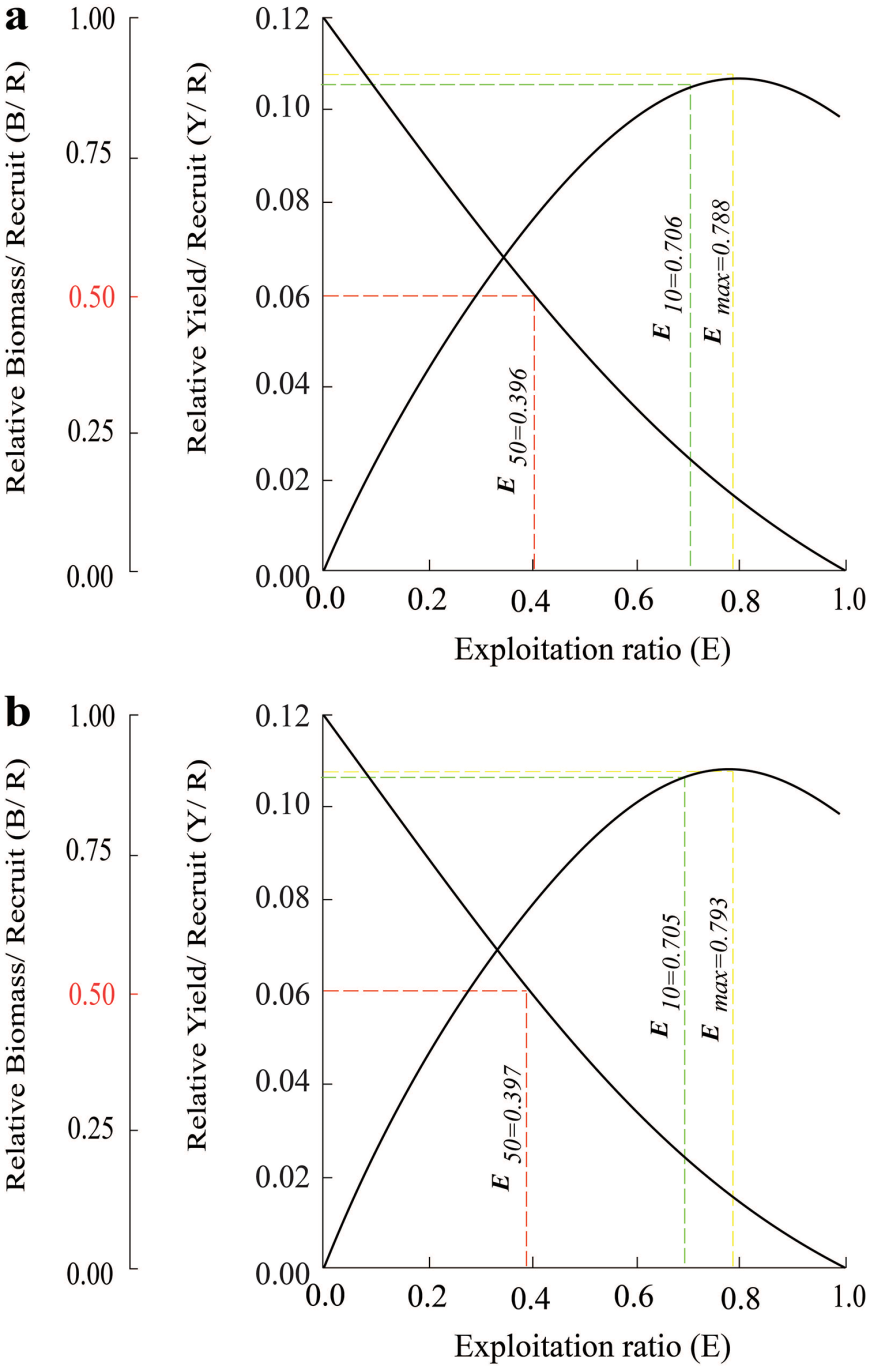
The relative yield-per-recruit (solid curve) and relative biomass-per-recruit (broken curve) using the knife-edge selection procedure of *Periophthalmus chrysospilos*. (A) Duyen Hai, Tra Vinh and Tran De, Soc Trang; (B) Dong Hai, Bac Lieu and Dam Doi, Ca Mau.

The survival rate gradually decreased from 3.0 cm to the 9.0 cm. Meanwhile, the total mortality rate increased across length groups. Similar results were found in the BLCM region, where the percentage of the fish group that survived over the fish group that died decreased, suggested that the size of fish was inversely proportional to their ability to survive (Fig. 6).

The effects of maximum exploitation rate (*E*_*max*_), optimal exploitation rate (*E*_*0.1*_), and the exploitation rate that the population decreased by 50% (*E*_*0.5*_) was 0.788, 0.706 and 0.396. These parameters in BLCM also had values of 0.793, 0.705 and 0.397, respectively (Fig. 6). The growth coefficient (*Φ′*) of the *P. chrysospilos* populations at TVST and BLCM were 1.83 and 1.79, respectively.

**Figure 7:**
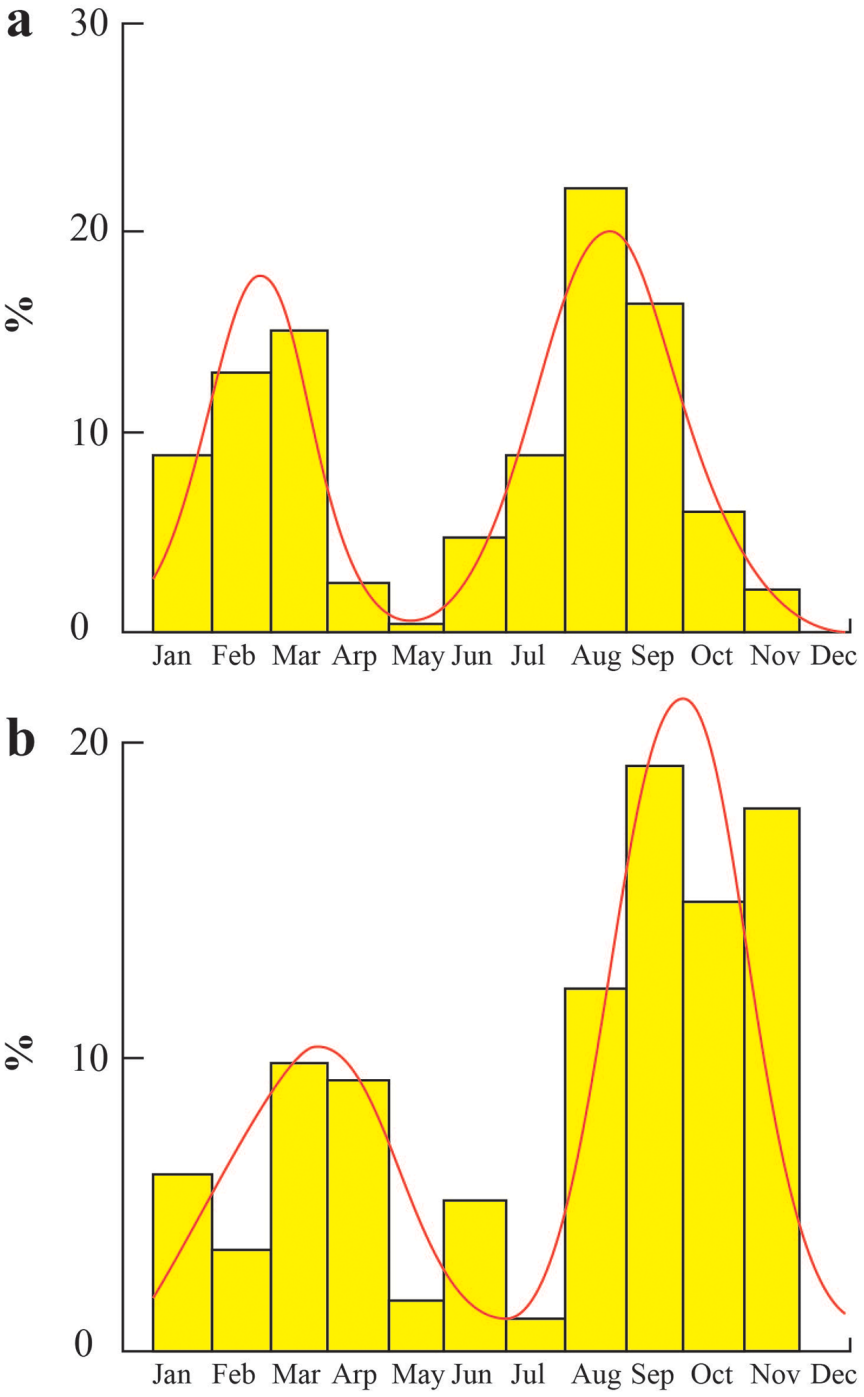
Population recruitment times of *Periophthalmus chrysospilos*. (A) Duyen Hai, Tra Vinh and Tran De, Soc Trang; (B) Dong Hai, Bac Lieu and Dam Doi, Ca Mau.

The time of population recruitment at TVST occurred twice a year in March and August (Fig. 7A), with rates of 15.15% and 22.08%, respectively. In BLCM, the population recruitment time took place in March and September with the rates of 9.56% and 19.47% (Fig. 7B). Besides the one-month delay, the population replenishment rate of BLCM was also lower than that of TVST.

## Discussion

Population parameters in *P. chrysospilos* showed differences between the two ecoregions in the VMD. In TVST, the fish displayed a significantly higher growth rate (*Φ′*) and maximum lifespan (*t*_*max*_), than in BLCM. Similarly, the exploitation coefficient (*E*) in TVST was also higher than that of BLCM. However, the large extraction coefficient should lead to a higher total mortality rate (*Z*) in the TVST population. The difference of these coefficients over the two ecoregions showed that the different environments had affected fish populations. With a more suitable environment in terms of phytoplankton (pH from 7.8–7.9, salinity 10.4–12.3% and a large mangrove cover), the population in this area developed better than in BLCM. But besides the influence of the environment on the growth rate and maximum lifespan of fish, environmental factors were seen to not affect other parameters such as maximum length (*L*_*∞*_), and the length of first exploitation (*L*_*c*_). Thereby, these coefficients are species-specific and less affected by the environment. This is verified by another study in Hong Kong which showed that the maximum length of this species was 12.9 cm (Kottelat et al., 1993)-roughly equivalent to the results in this study.

The sex ratios of *P. chrysospilos* in TVST (1.00:1.08) and BLCM (1.00:0.88) were equivalent to a ratio of 1:1. Approximately equal sex ratios have also been found for other mudskippers such as *Periophthalmodon schlosseri* (1.00:1.10) (Mazlan & Rohaya, 2008); *P. schlosseri* (1.16:1.00) (Tran et al., 2019); and *Boleophthalmus boddarti* (0.90:1.00) (Dinh, Nguyen & Nguyen, 2015). In *P. papilio* population in Nigeria, males dominated over females (1.00 : 0.70) (Lawson, 2010); contrary, males were less than females in *P. barbarus* (1.00:1.40) (Chukwu, Deekae & Gabriel, 2010). There were several possible reasons for this variation, including the quantity of caught males or females at random and the role changing between males and females to adapt to the different habitats (Dinh et al., 2020a).

Although within the same delta, in each different ecological zone, the fish displayed differing growth rate (*K*). The analysis results show that fish from TVST (0.41) had a faster growth rate than those of BLCM (0.38). This indicates that the environment in each region has a direct influence on this parameter. One of the crucial factors is the salinity and vegetation in these two ecoregions. It can be seen that *P. chrysospilos* had better growth ability in low salinity areas (≈10%) as in TVST. Compared to other fish species of the genus *Periophthalmus*, the mudskipper *P. chrysospilos* had a smaller growth parameter (*K*) (0.41 in TVST and 0.38 in BLCM). This *K* value is species- specific since this value is different in various species of the genus *Periophthalmus*. For example, the *K* values of *P. papillo* in Cross River, Nigeria (Etim, Brey & Arntz, 1996), *P. barbarus* in Nigeria (Etim, King & Udo, 2002), *P. novemradiatus* in Cox’s Bazar, Bangladesh (Rahman et al., 2015) and *P. waltoni* in Hormozgan Province, Persian Gulf (Sharifian et al., 2018) were 0.51, 0.55, 1.5 and 0.68, respectively. The *K* value of *P. chrysospilos* was lower than that of some mudskippers, *e.g*., *Boleophthalmus boddarti* in Mekong Delta (0.79) (Dinh, 2017), *P. septemradiatus* also in Mekong Delta (0.49) (Tran & Dinh, 2020), *P. schlosseri* in Malaysia (1.40) (Mazlan & Rohaya, 2008). It was recognized that *P. chrysospilos* had a slower growth rate than other species in the genus *Periophthalmus*. From the *K* values of *P. chrysospilos* and other species of *Periophthalmus* it can be understood that the *K* value can be specific to the species.

The environmental factors could affect fish growth coefficient (Φ′) (Pauly & Munro, 1984). Indeed, *Φ′* of this mudskipper in the TVST ecoregion (1.83) was higher than that of BLCM (1.79), due to the variation of pH and salinity between these ecoregions (Dinh et al., 2021a). Compared to its congeners distributing out of VMD, *Φ*′ of *P. chrysospilos* was lower than *P. papillo* (2.28) (Etim, Brey & Arntz, 1996), *P. barbarus* (2.41) (Etim, King & Udo, 2002), *P. novemradiatus* (1.91) (Rahman et al., 2015), *P. waltoni* (3.58) (Sharifian et al., 2018). It indicates that *Φ*′ of this genus showed specific species and spatial variation. The higher salinity in BLCM (23.2–23.5%), than in TVST (10.4–12.3%) led to earlier hatching time of *P. chrysospilos* in BLCM (~24 days; *t*_*0*_ = −0.08) than in TVST (~30 days; *t*_*0*_ = −0.10).

The relationship between the length of the first catch and the maximum size of fish played a role in determining the fishing age of each species. In this fish species, the *L*_*c*_*/L*_*∞*_ was about the same, 0.6, for both regions. These ratios were higher than that of some species of the genus *Periophthalmus*, such as *P. barbarus* in Nigeria (0.47) (Etim, King & Udo, 2002), and *P. novemradiatus* in Bangladesh (0.56) (Rahman et al., 2015). This difference may be influenced by environmental factors in the two regions. Salinity in the two study areas in VMD was quite low (10.4–12.3% in TVST and 23.2–23.5% in BLCM). Meanwhile, in two species, *P. barbarus* and *P. novemradiatus* distributed in Nigeria and Bangladesh, the average salinity was 21% (Etim, King & Udo, 2002) and 29% (Imran et al., 2020), respectively. The pH in these three regions also differed, with 7.6–7.9 in VMD, 6.8 in Nigeria (Etim, King & Udo, 2002) and 8.5 in Bangladesh (Imran et al., 2020). From these data, it can be seen that in the same genus, but with different environments, the age of fishing was also different. However, within the same area, the catching ages of some other oxudercinae fish living in the Mekong Delta were younger than *P. chrysospilos*, due to their lower *L*_*c*_*/L*_*∞*_, *e.g*., *Parapocryptes serperaster* (0.57) (Dinh, Qin & Tran, 2015), *P. septemradiatus* (0.55) (Tran & Dinh, 2020). Some other fish species in the Mekong Delta also had this ratio lower than *P. chrysospilos* such as *Pseudapocryptes elongatus* (0.45) (Tran et al., 2007), *Trypauchen vagina* (0.57) (Dinh, 2018b), *Butis butis* (0.44) (Dinh, 2018a), *Stigmatogobius pleurostigma* (0.44) (Dinh & Nguyen, 2018) and *B. koilomatodon* (0.44) (Dinh et al., 2020a). The difference in *L*_*c*_*/L*_*∞*_ ratios suggests that the most suitable age for catching is specific-species and affected by environmental conditions.

*Periophthalmus chrysospilos* displayed high fishing mortality, resulting from human activity affecting their habitat. However, the natural mortality rate of this fish is quite low compared to some other mudskipper species such as *P. barbarus* (1.35) (Etim, King & Udo, 2002), *P. novemradiatus* (3.39) (Rahman et al., 2015), *P. waltoni* (1.58) (Sharifian et al., 2018), *P. serperaster* (1.51) (Dinh, Qin & Tran, 2015), *P. septemradiatus* (3.14) (Tran & Dinh, 2020). This suggests that *P. chrysospilos* was inhabiting good environmental conditions.

The populations of *P. chrysospilos* in both ecoregions were not overfished because *E* was greater than *E*_*10*_. Specifically, in the TVST region, *E* = 0.64 was less than *E*_*10*_ = 0.706. At BLCM, these parameters were 0.53 and 0.705, respectively. This is different from some other fish species in the VMD that are being overexploited, such as *Glossogobius giuris* (Dinh, Phan & Tran, 2017), *B. butis* (Dinh, 2018a), *S. pleurostigma* (Dinh & Nguyen, 2018), *G. aureus* (Dinh, Tran & Tran, 2021dd), and *G. sparsipapillus* (Nguyen et al., 2021).

In conclusion, results show that *P. chrysospilos* could be an ideal candidate for aquaculture production and fishing because the population replenishment period took place twice a year in both two ecoregions. Although the population of this fish was not overexploited, its numbers were still decreasing. Therefore, it is necessary to protect mangrove forests, use favorable fishing gear with larger mesh sizes and avoid fishing at the time of the population recruitment for sustainable exploitation of this mudskipper.

## Supplemental Information

10.7717/peerj.13289/supp-1 Supplemental Information 1 Raw data.

## Additional Information and Declarations

### Data Availability

The following information was supplied regarding data availability: The raw data is available in the Supplemental File.
